# Recent advancements in bariatric/metabolic surgery

**DOI:** 10.1002/ags3.12030

**Published:** 2017-09-10

**Authors:** Wei‐Jei Lee, Owaid Almalki

**Affiliations:** ^1^ Department of Surgery Min‐Sheng General Hospital National Taiwan University Taoyuan Taiwan; ^2^ Department of Surgery College of Medicine Taif University Taif Saudi Arabia

**Keywords:** bariatric surgery, metabolic surgery, severe obesity, type 2 diabetes

## Abstract

Obesity and type 2 diabetes mellitus (T2DM) are currently two pan‐endemic health problems worldwide and are associated with considerable increase in morbidity and mortality. Both diseases are closely related and very difficult to control by current medical treatment, including diet, drug therapy and behavioral modification. Bariatric surgery has proven successful in treating not just obesity but also in significantly decreasing overall obesity‐associated morbidities as well as improving quality of life in severely obese patients (body mass index [BMI] >35 kg/m^2^). A rapid increase in bariatric surgery started in the 2000s when the laparoscopic surgical technique was introduced into this field. Many new procedures had been developed and changed the face of modern bariatric surgery. Recently, bariatric surgery played as gastrointestinal metabolic surgery has been proposed as a new treatment modality for obesity‐related T2DM for patients with BMI >35 kg/m^2^. Strong evidence has demonstrated that bariatric/metabolic surgery is an effective and durable treatment for obese T2DM patients. Bariatric/metabolic surgery is now becoming an important surgical division. The present article examines and discusses recent advancements in bariatric/metabolic surgery and covers four major fields: (i) the rapid increase in numbers and better safety; (ii) new procedures with better outcomes; (iii) from bariatric to metabolic surgery; and (iv) understanding the mechanisms and personalized treatment.

## 1 INTRODUCTION

Obesity and its related metabolic disorders are increasing to epidemic proportions at an alarming rate worldwide.^1^ It is estimated that more than 300 million adults worldwide are obese (body mass index ([BMI] >30 kg/m^2^). Obesity is a strong and independent risk factor for type 2 diabetes mellitus (T2DM), coronary heart disease, stroke, cancers and many other metabolic disorders, and is associated with increased mortality.^2^, ^3^, ^4^ Among all the obesity‐related comorbidities, T2DM boosted by the obesity epidemic has reached a pandemic level and is currently a significant challenge to the health‐care system worldwide. It is estimated that more than 415 million individuals were affected by T2DM worldwide in 2015 with a global prevalence of 8.8%.^5^ Furthermore, more than 60% of the world's population with diabetes comes from Asia and the incidence of T2DM in Asia is increasing more rapidly than in the rest of the world.^6^ Although the obesity prevalence in Asia is not high as in the Western world, Asia is in the epicenter of the T2DM epidemic.

How to control and treat this chronic and debilitating twin disease is currently a very important health problem. Unfortunately, current medical treatment has been relatively unsatisfactory in the treatment of obesity as well as T2DM.^7^, ^8^ Bariatric surgery, a weight reduction surgery, has been shown as not only an effective treatment for severe obesity (BMI >35 kg/m^2^) but has also resulted in marked improvement of T2D control.^9^, ^10^ Encouraged by the success of bariatric surgery, gastrointestinal metabolic surgery has been recently proposed as a new treatment modality for obesity‐related T2DM in patients with BMI <35 kg/m^2.^ 8, 11 Rapid development of bariatric/metabolic surgery for the treatment of obesity and T2DM has occurred in recent decades, although bariatric surgery has been introduced for more than 60 years. The present review summarizes recent advancements in bariatric/metabolic surgery and will build a foundation for updates in bariatric/metabolic surgery and for the development of further clinical trials to provide more evidence in this field for the treatment of obesity as well as T2DM.

## 2 INCREASE IN NUMBERS AND BETTER SAFETY

**Figure 1:**
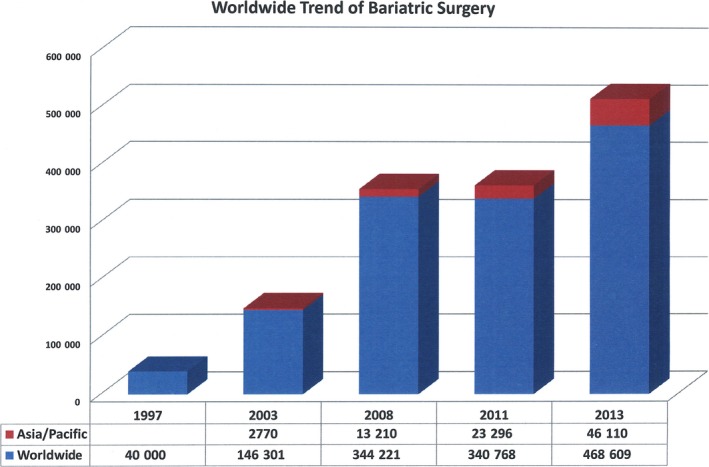
Total number of bariatric surgeries worldwide and in the Asia‐Pacific region according to statistics from International Federation for the Surgery of Obesity and Metabolic Disorders (IFSO)^13^, ^14^, ^15^, ^16^, ^17^

Bariatric surgery started from intestinal bypass in the USA more than 60 years ago in the 1950s and was abandoned after the development of vertical banded gastroplasty (VBG) and gastric bypass in the 1980s.^12^ With the development of laparoscopic surgery, bariatric surgery entered the realm of laparoscopic surgery, and laparoscopic VBG, gastric bypass and laparoscopic adjustable gastric banding (LAGB) were developed in the 1990s and emerged as an alternative to conventional bariatric surgery. Because laparoscopic surgery has increased the interest and growth of bariatric surgery, a soaring demand for laparoscopic bariatric surgery from patients has boosted the boom in bariatric surgery worldwide. According to the International Federation for the Surgery of Obesity and Metabolic Disorders (IFSO) survey, bariatric surgery has had more than 10‐fold growth worldwide in the past 20 years. The number of worldwide bariatric procedures increased from 40 000 bariatric procedures a year in 1997 to 468 609 in 2013.^13^, ^14^, ^15^, ^16^, ^17^ Owing to the high incidence of obesity, the bariatric procedure has become the most commonly carried out surgical procedure in the USA.^18^ However, it is interesting to see that the growth rate was higher in Asia than in other parts of the worldwide (Figure 1). In viewing the T2DM epidemic in Asia, bariatric/metabolic surgery will soon be a very important surgical division.

Although bariatric surgery, especially carried out by laparoscopic surgery, is one of the most common complex laparoscopic operations, the safety of laparoscopic bariatric surgery has improved very rapidly. The 30‐day operation mortality was reported to be 2% in 2004 and decreased to 0.2% in 2009 through the high‐quality bariatric center program in the USA.^19^, ^20^ Well‐experienced surgeons, fully trained in laparoscopic techniques and proctorship, teamwork and agdequate volume are important for a high‐quality bariatric surgical center.^21^ Improvements in technology, operative techniques, results of clinical trials and accumulation of experience all contributed to this progress.^22^ In the most recent publication, the 30‐day mortality from the European center of excellence program was reported to be only 0.012%.^23^ In conclusion, bariatric/metabolic surgery has had a 10‐fold growth in the past decade but the operation is 100‐fold safer now.

## 3 NEW PROCEDURES WITH BETTER OUTCOMES

**Figure 2:**
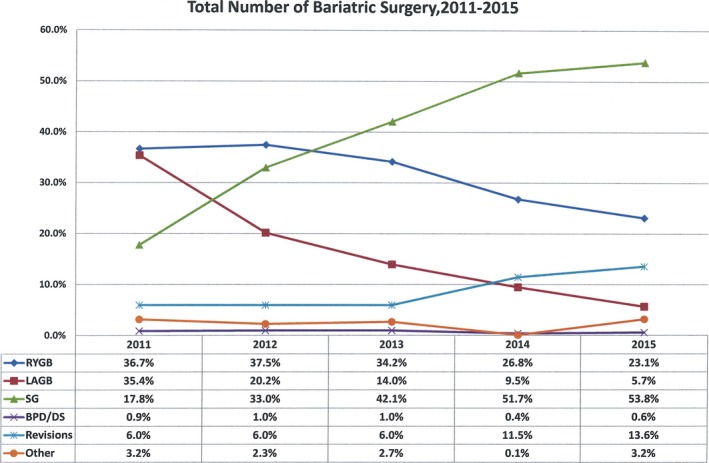
Total number of bariatric surgeries in the USA from 2011 to 2015 according to the statistics of American Society for Metabolic and Bariatric Surgery (ASMBS).^24^ BPD/DS, biliopancreatic diversion/duodenal switch; LAGB, laparoscopic adjustable gastric banding; RYGB, Roux‐en‐Y gastric bypass; SG, sleeve gastrectomy

Bariatric surgery has been evolving over the past 60 years and has undergone a significant increase in volume since the advent of laparoscopic surgery. However, the procedure of bariatric surgery is still evolving and numerous procedures with a plethora of variations are presently advocated as a method of choice to treat morbid obesity. Intestinal bypass and VBG have been abandoned and LAGB is severely decreasing.^12^ There has been a dramatic change in bariatric/metabolic procedures in the past decade. The most impressive change in bariatric procedure is the advent of laparoscopic sleeve gastrectomy (LSG). There is no reported case of LSG in the 2003 IFSO report.^14^ However, since 2014, LSG has become the leading bariatric procedure in the USA.^24^ Figure 2 shows the change of bariatric procedures in the USA from 2011 to 2015. According to the statistics of IFSO, the most commonly carried out procedure worldwide in 2011 was Roux‐en‐Y gastric bypass RYGB (45%), followed by LSG (37%) and LAGB (10%).^16^ Other procedures, such as laparoscopic single anastomosis (Mini‐) gastric bypass (LSAGB) and biliopancreatic diversion/duodenal switch (BPD/DS) comprise approximately 1.5% and 2.2%, respectively. Some of the commonly carried out procedures are introduced as follows.

### 3.1 Laparoscopic sleeve gastrectomy

Sleeve gastrectomy (SG) was first described as a first step as part of BPD/DS for obesity by Dr Doug Hess in March 1988.^25^ In the development of laparoscopic BPD/DS, Dr Gagner used LSG as a first‐stage bariatric procedure for high‐risk patients in order to reduce the risk profile of patients, and later carried out a second‐stage operation once adequate weight loss was achieved.^26^ However, it was noted that LSG alone could cause good weight loss before the second procedure.^27^ By removing approximately 75% of the stomach from the greater curvature side and leaving a long narrow gastric tube and antrum, LSG not only restricts food intake but also increases both gastric emptying and intestinal transit time. As a result of its effectiveness in weight loss, relative simplicity, and fewer long‐term nutritional problems, LSG has very rapidly now become the most commonly carried out bariatric/metabolic surgery worldwide.^16^, ^24^ LSG is especially welcomed by Asian surgeons for its advantages of avoidance of remnant gastric cancer.^28^

Laparoscopic sleeve gastrectomy was found to have a more durable and greater weight loss effect than other restrictive‐type operations such as VBG or LAGB.^29^ Total weight loss of approximately 25‐30% and excess weight loss of 60‐70% for up to 10 years can be achieved.^30^, ^31^ However, a significant drawback of this procedure is the development of reflux esophagitis. Up to 30% of LSG patients may require revision surgery for reflux esophagitis or weight regain.^29^, ^30^, ^31^, ^32^ Table 1 shows long‐term weight loss (>10 years) in different bariatric procedures.

**Table 1:** Ten‐year weight loss after different bariatric/metabolic procedures

	Case no.	Age (years)	BMI (kg/m^2^)	%TWL	%EWL	Revision rate %
LSG^30^	53	40.4	48.9	32.8	71	36
LSG^31^	65	38.7	38.8	23.6	67.4	25
LSG^32^	34	32.9	41.4	27.2	67.1	5.2
LRYGB^33^	65	41.5	47.8	NR	57.1	32.0
LRYGB^34^	46	35.4	38.5	26.7	60.1	5.1
LRYGB^35^	24	34.7	43.8	29.6	69	28.6
LRYGB^36^	40	37.9	44.5	NR	67.3	2.1
LRYGB^37^	134	42.9	46.6	31.6	58.9	23.5
LRYGB^38^	651	41.4	53.3	27.3	52.5	NR
LSAGB^34^	216	33.8	40.4	29.1	72.9	4.1
LAGB^39^	713	47.0	43.8	NR	47.3	43
LAGB^29^	109	43.2	35.7	14.7	38.4	19.3
LAGB^40^	718	39.5	45.6	NR	45.9	42.4
LAGB^41^	301	39.9	45.2	16.2	38.8	59.8
BPD^42^	37	37.1	44.3	40	76	13.1
BPD/DS^43^	56	42.3	51.8	39.0	67.9	37.0
LVBG^28^	20	44.4	40.7	20.1	42	14.3

Data are presented as mean (SD).

BMI, body mass index; BPD, biliopancreatic diversion; BPD/DS, biliopancreatic diversion/duodenal switch; EWL, excess weight loss; LAGB, laparoscopic adjustable gastric banding; LRYGB, laparoscopic Roux‐en‐Y gastric bypass; LSAGB, laparoscopic single anastomosis (Mini‐) gastric bypass; LSG, laparoscopic sleeve gastrectomy; LVBG, Laparoscopic VBG; NR, not reported; RYGB, Roux‐en‐Y gastric bypass; TWL, total weight loss.

### 3.2 Laparoscopic R‐Y gastric bypass

Five‐decades‐old gastric bypass surgery has become a time‐honored procedure and is currently regarded as a standard bariatric/metabolic procedure. Following the introduction of the laparoscopic era, laparoscopic R‐Y gastric bypass (LRYGB) has accelerated the development of both bariatric and metabolic surgery.^44^ Long‐term (>10 years) weight loss after RYGB was reported to be around 25‐30% total weight loss and 55‐70% excess weight loss **(**EWL).^33^, ^34^, ^35^, ^36^, ^37^, ^38^ Up to 20% of RYGB patients may require revision surgery for various complications or weight regain. Weight regain after LRYGB was related to dilatation of the gastric pouch and anastomosis.^45^, ^46^ Endoscopic treatment was developed recently and is recommended for first‐line treatment for those with weight regain after RYGB.^47^, ^48^ Weight loss after LRYGB was found to be similar or slightly better than with LSG but with more nutritional deficiencies in LRYGB.^49^, ^50^, ^51^ Hence, LSG is now becoming the leading bariatric/metabolic procedure instead of LRYGB.

### 3.3 Laparoscopic single anastomosis (Mini‐) gastric bypass

A simplified single anastomosis gastric bypass, LSAGB or Mini‐Gastric Bypass, was first reported by Rutledge in 2001.^52^ Although some controversy concerning the procedure existed in the USA, one randomized study confirmed it is a simpler and safer procedure compared to LRYGB.^53^ Many other reports of large series confirmed the safety and long‐term efficacy of this procedure.^54^, ^55^, ^56^, ^57^ LSAGB can also be used in revision surgery for failed restrictive‐type procedures with good results.^58^, ^59^ Long‐term (>10 years) weight loss after LSAGB was reported to be around 30% total weight loss and 70‐75% EWL.^34^, ^52^ However, this procedure has an increased risk of malnutrition because the bypass limb is longer than in RYGB.^34^, ^52^ Up to 5% of LSAGB patients may require a revision surgery for malnutrition, weight regain and complications.^60^, ^61^

### 3.4 Laparoscopic adjustable gastric banding

LAGB is the safest bariatric surgical procedure but the efficacy is less favored than other bariatric procedures. Although some reports had good results after LAGB, most of the reports had less favor weight loss and a high revision rate.^39^, ^62^ After the emergence of LSG, LAGB was replaced by LSG rapidly in almost every part of the world.^28^, ^30^ Since 1999, Asian countries also started to carry out LAGB, first in Singapore, then in Taiwan, China, Hong Kong and India. However, our experience in Asian patients disclosed that although LAGB was successful in weight loss and in resolution of comorbidities in morbidly obese patients, the gastrointestinal quality of life index (GIQLI) did not improve and the long‐term revision rate was high.^63^ Long‐term (>10 years) weight loss after LAGB was reported to be around 15% total weight loss and 40‐45% EWL.^31^, ^40^, ^41^, ^62^ However, up to 50% of LAGB patients may require revision surgery to another procedure for inadequate weight loss.^39^, ^41^, ^62^, ^63^

### 3.5 Laparoscopic biliopancreatic diversion/duodenal switch

Biliopancreatic diversion (BPD), a procedure that combines distal gastric resection and intestinal malabsorption, was introduced by Scopinaro in 1976.^42^ Then, Hess et al. and Marceau^25^ modified the procedure by replacing the distal gastrectomy with a sleeve gastrectomy and preserving the pylorus (known as BPD/DS).^25^ This procedure is the most powerful bariatric surgery in weight loss and has improvement in comorbidities. However, significant malabsorption complications and difficulty in laparoscopic surgery prevent the wide usage of this procedure.^17^ Long‐term (>10 years) weight loss after BPD/DS was reported to be around 40% total weight loss and 80% EWL.^42^, ^43^ Revision surgery was required in 37% of the patients.^43^

### 3.6 New procedures

Many new bariatric/metabolic surgeries have been introduced in the past decade. However, most of the procedures were experimental, and there is lack of evidence and long‐term data. The most interesting procedure was duodenojejunal bypass (DJB), which was inspired by Rubino's animal experiment for the treatment of T2DM.^64^ However, simple exclusion of the duodenum was found to be less effective than conventional bariatric/metabolic surgery.^65^ Therefore, duodenojejunal bypass with sleeve gastrectomy (DJB‐SG) has recently been introduced from Asia as a novel metabolic surgery by adding a duodenal switch procedure to SG, which combines the principles and advantages of sleeve gastrectomy and duodenal switch.^66^ The efficacy of DJB‐SG was similar to conventional gastric bypass.^28^ The major advantage of DJB‐SG compared to RYGB is avoidance of the risk of gastric cancer arising from the remnant stomach by leaving no excluded stomach. Other theoretical advantage of DJB‐SG is related to preservation of the pylorus, which includes prevention of dumping syndrome and facilitating iron, calcium, vitamin B12 and protein absorption by preserving the acid and intrinsic factor.^67^ A recent study showed that by adding a duodenal exclusion to SG, DJB‐SG can increase weight loss by more than 10% and improve glycemic control as well as reduce uric acid level.^68^ This finding further supported the important role of duodenum exclusion in the treatment of T2DM. A five‐year report was published recently from Japan to support the efficacy of this procedure.^69^

Another interesting new procedure was single anastomosis duodeno‐ileostomy (SADI), a simplified BPD/DS using a loop anastomosis replacing the RY anastomosis of the duodenal switch.^70^ Similar modification was laparoscopic single anastomosis for DJB (LSADJB‐SG).^28^ Both procedures are a simplified version of the original operation by replacing the RY reconstruction by loop anastomosis, as is SAGB to RYGB.

Other novel procedures, such as LSG with ileal transportation,^71^ LSG with proximal jejunal bypass,^72^ LSG with bi‐partition,^73^ laparoscopic greater curvature side gastric plication,^74^, ^75^ banded gastric plication 76 and Nissen fundoplication with gastric placation 77 were either too complicated or without enough evidence to be supported in clinical use.

However, there is a trend of moving from laparoscopic bariatric/metabolic surgery to endoscopic procedures.^78^ Most commonly carried out endoscopic procedure was intragastric balloon.^79^ Duodenojejunal sleeve liner was developed under the concept of foregut theory proposed by Rubino.^80^ It is a thin flexible 60‐cm‐long tube that is delivered endoscopically and creates a physical barrier between ingested food and the duodenum/proximal jejunum. Endoscopic suturing technique was applied in the endoscopic sleeve 81 or for salvage purposes.^42^, ^43^

## 4 FROM BARIATRIC TO METABOLIC SURGERY

Type 2 diabetes mellitus, fueled by an obesity epidemic, has emerged as a major health problem worldwide. Initiation of bariatric surgery for the treatment of T2DM started from the report by Pories et al. in 1995.^82^ Strong evidence has shown that bariatric surgery is an effective treatment for severe obesity (BMI >35 kg/m^2^) and results in marked improvement of T2DM control.^10^, ^83^, ^84^ Derived from bariatric surgery, metabolic surgery is focused on T2DM treatment in mildly obese or overweight patients (BMI <35 kg/m^2^).^85^ From Asia, in 2004, Lee et al. published the first paper in the world to report the effectiveness of bariatric surgery on the treatment of metabolic syndrome.^86^ In 2008 and 2009, Lee et al. then published the first two reports on the efficacy of bariatric/metabolic surgery on T2DM treatment in Asians.^87^, ^88^ Asian Pacific Metabolic and Bariatric Surgery Society (APMBSS) was the first society to propose using bariatric/metabolic surgery for T2DM treatment and, in 2005, recommended setting the indication for metabolic surgery at a lower BMI of 32 kg/m^2^.^89^ The first randomized controlled trial (RCT) in the world specific to metabolic surgery for the treatment of T2DM was from Asia.^90^ This study compared LSG to gastric bypass (LSAGB) for the treatment of T2DM in Asian patients with BMI <35 kg/m^2^ and was the first study to prove that gastric bypass (a duodenum exclusion procedure) had better efficacy on T2DM remission than LSG (non‐duodenum exclusion procedure). Following this landmark study, many RCT focusing on the comparison of metabolic surgery and medical treatment for the treatment of T2DM have been carried out and universally showed that metabolic surgery is more effective than medical treatment in glycemic control.^91^, ^92^, ^93^, ^94^, ^95^, ^96^, ^97^, ^98^, ^99^ Table 2 lists the results of RCT trials on surgical treatment versus medical treatment for T2DM. However, because of their small number of cases and short‐term follow up, none of these studies has demonstrated a survival benefit and reduction of T2DM‐related clinical end‐organ damage. The potential benefits of metabolic surgery to prevent mortality and end‐organ damage may only be confirmed by large multicenter trials with long‐term follow up.

**Table 2:** Randomized trials comparing bariatric/metabolic surgery with medical treatment for T2DM

Author	Year	No. cases	Mean BMI, kg/m^2^ (range)	Mean HbA1c %	Follow up (years)	Post‐treatment HbA1c %	T2DM remission %
Dixon^91^	2008	Medical (30) LAGB (30)	36 (30‐40)	7.8	2	Medical (7.2) LAGB (6.0)	Medical (13)^b^ LAGB (73)
Liang^92^	2013	Medical (77) RYGB (31)	30 (28‐35)	10.5	1	Medical (8.0) RYGB (6.0)	Medical (6)^d^ LAGB (90)
Wentworth^93^	2014	Medical (26) LAGB (25)	29 (25‐30)	6.9	2	Medical (7.1) LAGB (6.4)	Medical (4)^d^ LAGB (52)
Courcoulas^94^	2014	Medical (20) RYGB (21) LAGB (21)	35.5 (30‐40)	7.9	1	Medical (6.9) RYGB (6.4) LAGB (6.9)	Medical (0)^c^ RYGB (50) LAGB (23)
Halperin^95^	2014	Medical (19) RYGB (19)	36.3 (30‐40)	8.5	1	Medical (8.4) RYGB (6.7)	Medical (16)^c^ RYGB (58)
Mingrone^96^	2015	Medical (20) RYGB (20) BPD (20)	48.7 (>35)	8.7	5	Medical (6.9) RYGB (6.7) BPD (6.4)	Medical (27)^c^ RYGB (42) BPD (68)
Cummings^97^	2016	Medical (17) RYGB (15)	37.8 (30‐45)	7.5	1	Medical (6.9) RYGB (6.4)	Medical (5.9)^c^ RYGB (60)
Schauer^98^	2017	Medical (50) LSG (50) RYGB (50)	36 (28‐42)	9.4	5	Medical (8.5) LSG (7.4) RYGB (7.3)	Medical(5.3)^a^ LSG (20.4) RYGB (26.4)
Ikramuddin^99^	2017	Medical (60) RYGB (60)	34.6 (30‐40)	9.6	5	Medical (8.6) RYGB (6.7)	Medical (7)^a^ RYGB (27)

^a^A1c <6.0%; ^b^A1c <6.2%; ^c^A1c <6.5%; ^d^A1c <7.0%.

BPD, biliopancreatic diversion; BMI, body mass index; HbA1c, hemoglobin A1c; LAGB, laparoscopic adjustable gastric banding; LSG, laparoscopic sleeve gastrectomy; RYGB, Roux‐en‐Y gastric bypass; T2DM, type 2 diabetes mellitus.

## 5 UNDERSTANDING THE MECHANISM

One of the major advancements in bariatric/metabolic surgery is understanding the mechanism. Bariatric/metabolic surgery is a gastrointestinal surgery and its effect is through various gastrointestinal anatomical changes and reroute.^100^ Updated theory of mechanism is summarized below.

### 5.1 Gastric restriction

Procedures without gastric restriction, such as intestinal bypass or duodenal bypass, can produce only a minimal weight loss.^101^ VBG was the first successful bariatric procedure with a pure gastric restriction effect. LAGB is another pure gastric restrictive procedure. Both procedures can provide an average of approximately 15% total weight loss in the long term, but many patients require revision for weight regain. The gastric restrictive effect of gastric bypass was provided by a small gastric pouch and small gastrojejunal anastomosis. Weight regain after gastric bypass was commonly attributed to dilated gastric pouch and wide anastomosis.^40^, ^41^ Proposed management of weight regain after gastric bypass was resizing the gastric pouch or endoscopic downsizing of the gastrojejunostomy. 42, 43 Therefore, gastric restrictive effect was considered to be the most important part of metabolic surgery and comprised about 70% of the effect of gastric bypass.^17^

### 5.2 Exclusion of duodenum and upper intestine

Rerouting the gastrointestinal (GI) tract by Roux‐en‐Y reconstruction causes exclusion of the duodenum and upper part of the jejunum from exposure to ingested nutrients. This anatomical change may alter the physiological response of digestive enzyme secretion from the duodenum, gut hormone changes and nutrient sensing of the upper small intestine.^102^, ^103^ For example, DJB was a procedure to exclude the duodenum and proximal jejunum without gastric restriction, and improved glycemic control without reduction of food intake and weight loss.^65^ A recently developed new device, duodenum jejunal sleeve liner, also had a similar effect to DJB.^80^

### 5.3 Rapid delivery of food or short common channel

Rerouting the GI tract by gastric bypass not only excludes the duodenum but may rapidly deliver incompletely digested food to the distal bowel which may induce a strong gut hormone change, mainly peptide tyrosine tyrosine (PYY) and glucagon‐like peptide 1 (GLP‐1). Interestingly, sleeve gastrectomy was found to have this effect without rerouting the GI tract possibly as a result of rapid intestine transit time.^104^

### 5.4 Gut microbiota environment and bile acid metabolism

Obese patients were found to have different microbiota that can be changed after bariatric surgery.^105^ In addition, microbiota transfer from lean mice has a weight‐reducing effect on obese mice.^106^ However, a more important mechanism than microbiota was the finding of bile acid mechanism. Serum bile acid was found to increase after bypass surgery but not in LAGB, and the increase was correlated with weight loss.^107^ Recent experiments on gene kick‐out mice demonstrated that bile acid, not GLP‐1, was the key player in SG.^108^ Therefore, bile acid, through the nuclear farnesoid X receptor (FXR) in liver and small intestine, may play very important roles in weight reduction and T2DM remission.^109^

In conclusion, the underlying mechanism for bariatric/metabolic surgery is intriguing and many theories were recently proposed but none of these theories necessarily precludes the others. Further studies are mandatory to elucidate the mechanism of bariatric/metabolic surgery. Through this knowledge, new treatment modality or novel medicine may thus be developed.

## 6 PERSONALIZED TREATMENT

Optimal outcomes for bariatric/metabolic surgery can be achieved if patients best suited to the surgery are selected and those who will predictably have a poor result are excluded. In addition, we also need to choose a best operation procedure for the patient because now there are so many available bariatric/metabolic surgical procedures. To be able to make such decisions, we need long‐term data, evidence from comparative studies and predictors of success or failure. Recent advancements in clinical research and understanding of the mechanism in bariatric/metabolic surgery has paved the way for precision medicine in this field, a personalized treatment of bariatric/metabolic surgery. For example, LSG may be the first choice of bariatric procedure but should be avoided in patients with reflux disease.^30^, ^31^ RYGB is a better choice for morbidly obese patients with significant reflux disease. For diabetic patients, RYGB or LSAGB may be a better choice for T2DM patients, especially in patients with low BMI and long duration of disease.^92^, ^110^ However, SG‐DJB might be a better choice for patients from a gastric cancer endemic area or with a family history of gastric cancer. Patients super‐morbidly obese (BMI >50) may consider a malabsorptive procedure, BPD/DS or LSAGB.^111^ However, the patients should be notified about the risk of malnutrition and must take supplements.

For T2DM patients, primary endpoint and surgical risk are different with severely obese patients without metabolic syndrome or T2DM.^17^, ^112^ Current indications for bariatric obesity surgery are based on BMI and metabolic surgery is recommended for T2DM Asians with BMI >27.5 kg/m^2^.^11^ Although T2DM remission is closely associated with BMI, many other factors, such as abdominal obesity, β‐cell function, duration of disease and age are all important predictors of T2DM remission after metabolic surgery.^113^ To combine these important predictors, a scoring system consisting of four variables: age, BMI, C‐peptide level and duration of diabetes has been developed.^114^ This ABCD Diabetes Surgery Score system is especially designed for predicting the success of metabolic surgery and has been validated in many studies.^115^, ^116^, ^117^ In clinical practice, this scoring system can help the endocrinologist to set the priority for referring patients for metabolic surgery and the surgeon to counsel patients for metabolic surgery and choice of surgical procedure in the construction of personalized treatment.

## 7 CONCLUSIONS

The success of bariatric surgery in severely obese individuals (BMI >35 kg/m^2^) has led to a paradigm shift of metabolic surgery for the treatment of T2DM, including patients with a BMI <35 kg/m^2^. A rapidly increasing demand for bariatric/metabolic surgery has been noted worldwide. The mechanisms of bariatric/metabolic gastrointestinal surgery are thought to be dependent on the dramatic enterohormonal changes after physio‐anatomical rearrangement of the gastrointestinal tract. Accumulation of knowledge about the mechanism and differences between procedures has paved the way for individual treatment planning. Further progress of bariatric/metabolic surgery in the future depends on the elucidation of the effect of gut hormone and neuroendocrine mechanisms as well as individual gene polymorphism. How to provide safe bariatric/metabolic surgery, train qualified bariatric surgeons and continue to develop better techniques will be important issues in the surgical treatment of obesity in the future.

## DISCLOSURE

Conflict of Interest: Authors declare no conflicts of interest for this article.
